# Targeting Stress Sensor Kinases in Hepatocellular Carcinoma-Infiltrating Human NK Cells as a Novel Immunotherapeutic Strategy for Liver Cancer

**DOI:** 10.3389/fimmu.2022.875072

**Published:** 2022-05-23

**Authors:** Alessandra Zecca, Valeria Barili, Andrea Olivani, Elisabetta Biasini, Carolina Boni, Paola Fisicaro, Ilaria Montali, Camilla Tiezzi, Raffaele Dalla Valle, Carlo Ferrari, Elisabetta Cariani, Gabriele Missale

**Affiliations:** ^1^ Unit of Infectious Diseases and Hepatology, Laboratory of Viral Immunopathology, Azienda Ospedaliero–Universitaria of Parma, Parma, Italy; ^2^ Department of Medicine and Surgery, University of Parma, Parma, Italy; ^3^ Independent Researcher, Modena, Italy

**Keywords:** immunometabolism, NK-cell, immunotherapy, hepatocellular carcinoma, oncoimmunology

## Abstract

Natural killer (NK) cells may become functionally exhausted entering hepatocellular carcinoma (HCC), and this has been associated with tumor progression and poor clinical outcome. Hypoxia, low nutrients, immunosuppressive cells, and soluble mediators characterize the intratumor microenvironment responsible for the metabolic deregulation of infiltrating immune cells such as NK cells. HCC-infiltrating NK cells from patients undergoing liver resection for HCC were sorted, and genome-wide transcriptome profiling was performed. We have identified a marked general upregulation of gene expression profile along with metabolic impairment of glycolysis, OXPHOS, and autophagy as well as functional defects of NK cells. Targeting p38 kinase, a stress-responsive mitogen-activated protein kinase, we could positively modify the metabolic profile of NK cells with functional restoration in terms of TNF-α production and cytotoxicity. We found a metabolic and functional derangement of HCC-infiltrating NK cells that is part of the immune defects associated with tumor progression and recurrence. NK cell exhaustion due to the hostile tumor microenvironment may be restored with p38 inhibitors with a selective mechanism that is specific for tumor-infiltrating—not affecting liver-infiltrating—NK cells. These results may represent the basis for the development of a new immunotherapeutic strategy to integrate and improve the available treatments for HCC.

## Introduction

Hepatocellular carcinoma (HCC) is the most frequent type of primary liver cancer with high incidence and mortality rate (1, 2). The very early and early stages of HCC can be cured by surgery and/or loco-regional therapies. However, in the advanced stage, only a limited choice of effective strategies exists, and tumor recurrence can be very aggressive (1). Similarly to other cancers, immunotherapy will become a central strategy also for HCC (3). Despite the fact that immunotherapy has become the first-line treatment for several cancers in recent years (4, 5), the first immunotherapy for HCC, based on anti-PD-L1 in association with anti-VEGF, has been registered only last year after the failure of trials based on a single anti-PD-1 treatment (6).

Natural killer (NK) cells account for 25–50% of lymphocyte populations infiltrating the liver (7). Intrahepatic NK cells play a central role in innate immune response against liver pathogens and tumors (8–10). The results in lung cancer suggest that NK cells entering the tumor microenvironment display markers of activation and cytotoxicity but become rapidly exhausted after target recognition (10). Even though available data support the role of NK cells in the control of HCC progression, there is evidence of the rapid functional exhaustion of NK cells in the HCC microenvironment which is associated with tumor progression and poor clinical outcome (11–16). This suggests that NK cell-based immunotherapies may enhance the NK cell effector function. Among immunotherapeutic approaches, checkpoint blockade and adaptive transfer of cytokine-activated killer cells have demonstrated some clinical efficacy (17, 18). However, the limited knowledge of the mechanisms of NK cell exhaustion has not allowed the use of NK cell immunotherapy in HCC.

NK cell activation is regulated by the balance between inhibitory and activating signals, which determines whether an NK cell will be able to kill a target (19). Moreover, NK function is strongly influenced by the surrounding cytokine environment and by the interaction with other cells, such as regulatory T cells, myeloid-derived suppressor cells, and tumor-associated macrophages (20). NK cell activation is characterized by a high metabolic activity and upregulation of OXPHOS and glycolysis, with enhanced transport of nutrients, in particular glucose transporter, to support antitumor functions (21, 22).

A tumor is a hostile microenvironment for immune cells due to hypoxia, nutrient deprivation, and release of immunosuppressive cytokines such as TGF-β (23). The low oxygen concentrations induce the tumor cells to switch from aerobic metabolism to anaerobic glycolysis, which exploits most of the available glucose (24).

HCC is a highly hypoxic tumor showing a particularly low median oxygen level (23, 25). The dramatic increase in glucose metabolism in tumor cells leads to decreased glucose availability and a dramatic increase in lactate level, which accumulates in the local microenvironment and induces the metabolic deregulation of infiltrating immune cells such as NK cells (22, 23).

Hypoxia and high intracellular lactate induce the dysfunction of hepatic cytotoxic lymphocytes, downregulating activating receptors and cytotoxic molecules or inducing apoptosis through reactive oxygen species (ROS) accumulation and mitochondrial damage, particularly in CD56^BRIGHT^ NK cell subpopulation (21, 26, 27). In addition, TGF-β contributes to corroborate the glycolytic impairment. It has been shown, in lung cancer, that the aberrant expression of FBP1, an enzyme involved in gluconeogenesis, due to TGF-β and glucose depletion can inhibit NK cell glycolysis, thus determining ROS accumulation (28). Moreover, the combined effect of TGF-β and hypoxia can determine the excess of mitochondria fragmentation *via* fission, followed by decreased OXPHOS and an increase of ROS levels (28–30). In metastatic breast cancer patients, TGF-β blockade rescued NK cells from impaired glycolysis and mitochondrial respiration (31, 32). In the mouse model of murine cytomegalovirus infection, NK cells accumulate dysfunctional mitochondria and activate mitophagy as a rescue mechanism, thus facilitating memory formation (33). The role of TGF-β in shaping the NK cell metabolic rearrangement was also shown in the peripheral blood of patients with HCC, where TGF-β-specific targeting could partially restore the NK cell dysfunctions (34).

The opportunity of restoring NK cell function by targeting NK cell immunometabolism has become an active area of research in oncoimmunology (28, 35, 36). In this complex scenario, we may speculate that drugs modulating immune metabolism could provide new therapeutic strategies by enhancing the NK cell response against HCC.

To better define and validate the key dysregulated pathways associated with NK cell exhaustion in HCC, we applied genome-wide transcriptome profiling of infiltrating NK cells and targeted rescue strategies with functional and metabolic validation. Here we show that HCC-infiltrating NK cells are marked by a predominantly upregulated gene expression profile translating into the metabolic and functional impairment of glycolysis, OXPHOS, and autophagy. Targeting dysfunctional signaling *in vitro* can efficiently improve the functions of HCC-infiltrating NK cells and may thus represent a novel strategy to be implemented in HCC treatment.

## Materials and Methods

### Patients and Biological Samples

The enrolled patients were featured by a class A liver cirrhosis (Child–Pugh) and an early stage of HCC (Barcelona Clinic Liver Cancer Stage A) diagnosed through ultrasonography/computed tomography or MRI in specific cases. All the patients were HBV- and HIV-negative. Additional information on the study population are shown in **Supplementary Table S1**.

Tumor and non-tumorous specimens were obtained from 11 HCC patients infected with hepatitis C virus, 6 patients with alcohol-associated HCC, one NASH and HCC patient, and control samples from the liver resection of 7 patients with colorectal metastasis. Functional and metabolic analyses of tissue-infiltrating NK cells were conducted on 12 matched HCC–non-tumorous samples and controls. Eight paired HCC– non-tumorous samples were used to evaluate the effect of *in vitro* treatment on cell metabolism.

The study underwent local ethical committee approval [Comitato Etico Indipendente Area Vasta Emilia Nord (AVEN) of the AOU of Parma, Parma, Italy]. Signed informed consent for each patient was obtained for them to take part in the study.

### Gene Expression Profiling

Thawed liver- and tumor-infiltrating lymphocytes from the 11 HCC and HCV patients and from the 6 liver-resected control patients were stained with anti-CD3-PeCy7 (Biolegend, San Diego, CA, USA), anti-CD16 FITC (BD Bioscience, Franklin Lakes, NJ, USA), anti-CD56 PECF594 (BD), and then 7-amino-actinomycin D [7AAD PerCPCy5.5 vitality dye, for discriminating NK cells in normal liver-infiltrating NK cells (NLINK), tumor-infiltrating NK cells (TINK), and NK cells infiltrating hepatic tissue around HCC (LINK)]. NK cell sorting was performed with FACSAria III Cell Sorter (BD) by selecting CD3-negative, CD56-positive, CD16-positive, or CD16-negative subpopulations.

RNA was extracted from the isolated NK cells with the RNase Free DNase I Kit (Norgen Biotek), and the RNA concentration was measured using a Nanodrop spectrophotometer, in accordance with the manufacturer’s instructions. The RNA integrity was detected with a Bioanalyzer2100 system (Agilent Technologies). Transplex Whole Transcriptome Amplification (WTA2) and GenElute PCR Clean-Up kits (Sigma) were employed to amplify and then purify the total RNA, respectively, following the manufacturer’s protocol. cDNA labeling was performed by SureTag DNA Labeling kit (Agilent Technologies) and then hybridized to 60-bp oligonucleotide whole human genome arrays (Human GE 8x60K v2 SurePrint G3, Agilent Technologies), following the manufacturer’s instruction. The microarrays were scanned with an Agilent dual-laser DNA scanner. The Agilent Feature Extraction software, v.7.5, was employed with default settings to achieve normalized expression values from raw data. The expression data are available at the National Center for Biotechnology Information Gene Expression Omnibus: GSE183349.

### Microarray Statistical Analysis

GeneSpring software package GX 13.1 (Agilent Technologies) was employed for quality control, data normalization (75th percentile procedure), and preliminary microarray data analysis. The probe signals in at least four replicates for each condition were kept for subsequent analyses. Benjamini–Hochberg-corrected ANOVA for multiple testing [false discovery rate (FDR), ≤0.05] was employed to track differentially expressed genes (DEGs) by comparing LINK-, TINK-, and NLINK-derived samples. Student–Newman–Keuls *post*-*hoc* test was used to determine the DEGs in the three patient groups.

Median baseline transformation was applied before determining the unsupervised hierarchical clustering of the samples with Ward’s linkage and Euclidean distance. In order to reduce data dimensionality, principal component analysis (PCA) was used with orthogonal transformation to cluster sample populations.

Gene Set Enrichment Analysis (GSEA) was employed on the detected probes in order to pinpoint significant pathways enriched in downregulated and upregulated probes and to analyze the gene profiles with other studies. Molecular Signature Database C2, canonical pathways version 6, was employed for statistical analysis with the permutation type (“gene set” for fewer than seven replicates); the default settings for all other options were kept. Significantly enriched gene sets were obtained with a FDR lower than 0.25, which was determined using 1,000 permutations of gene set and Signal2Noise as metric.

### Assessment of Infiltrating NK Cell Metabolic Features

For all flow cytometry analysis, after the exclusion of cell aggregates, gating was performed on lymphocytes, and dead cells were subsequently excluded (7-AAD or live/dead as appropriate); CD3, CD56, and CD16 were used to select NK cells that were identified as CD3− CD56+.

Mitochondrial membrane potential was detected on infiltrating NK cells through the JC-1 probe to measure the mitochondrial membrane potential (ThermoFisher). Anti-CD3 APCCy7 and anti-CD56 APCR700 were used to stain LINK, TINK, and NLINK, and then the cells were treated for 10 min at RT with JC-1 (2.5 μg/ml) before the FACS analysis. Finally, viability probe 7-AAD was used for cell staining, and FACS Canto II was used to acquire samples. Mitochondrial depolarization was measured in NK cells by quantifying the percentage of FL1high/FL2low cells (JC-1 staining) detected in the different samples.

The uptake of fluorescent glucose was assessed in order to define the glycolysis capacity. After washing with 1× phosphate-buffered saline (PBS) to withdraw endogenous glucose, the infiltrating lymphocytes were marked for 30 min at 37°C with the analog 2-NBDG {2-deoxy-2-[(7-nitro-2,1,3-benzoxadiazol-4-yl) amino]-D-glucose, ThermoFisher} at 40 μM in RPMI without glucose and added with 10% dialyzed fetal bovine serum, and then staining was caried out with anti-CD3, anti-CD56, and 7-AAD probes. The frequency and median fluorescence intensity (MFI) of 2-NBDG NK-positive cells were measured.

The autophagy potential was analyzed through the Cyto-ID Autophagy kit (Enzo Life Sciences, NY, USA) that detects autophagic vesicles and reveals autophagic flux in chloroquine diphosphate treatment to inhibit lysosomes and live cells (overnight at 30 µM), through a probe, which accumulate within the autophagic vacuole.

Changes in Cyto-ID expression were measured with or without chloroquine-mediated blocking of the autophagosome turnover, which hampers the fusion of autophagosome–lysosome vesicles, thus preventing their degradation.

On the following day, the NK cells were washed and resuspended in 1× PBS in order to eliminate any trace of phenol red. The Cyto-ID Autophagy kit was employed after anti-CD3 and anti-CD56 staining, according to the manufacturer’s instructions, for the final acquisition on a FACS Canto II flow cytometer. Data were expressed as Cyto-ID MFI in NK cells from different groups.

### Phosphorylation Status of p38 Protein in Infiltrating NK Cells

After thawing, the liver- and tumor-infiltrating NK cells (7 NLINK, 12 LINK, and 12 TINK) were stained with surface antibodies (anti-CD3-Alexa Fluor700 and anti-CD56-PerCp). Then, for anti-phosphorylated-p38 assay, Fixation/Permeabilization Solution Kit (Cytofix/Cytoperm BD) for intracellular staining was employed, in accordance with the manufacturer’s protocol and the antibody specific for the phosphorylated form of the human p38 mitogen-activated protein kinase (MAPK; pT180/pY182)PE-Cy7 (BD). Samples were acquired with a FACSCANTO II BD and analyzed with DIVA (BD) and Flowjo software. Data were expressed as phosphorylated-p38 MFI in NK cells from different groups.

### Functional Analysis of Infiltrating NK Cells

IFNγ and TNFα cytokine protein expression in NK cells was assessed after 4 h of phorbol 12-myristate 13-acetate (PMA, 50 ng/ml) and ionomycin (1 µg/ml) stimulation. After 1 h, Brefeldin A (BFA, 10 µg/ml) was supplemented. Anti-CD3 PE (BD), anti-CD56 PECF594 (BD), and anti-CD16 FITC (BD) were used to stain the cells before fixing with medium A reagent and then permeabilization with medium B reagent (Nordic Mubio), according to manufacturer’s instructions. Cytokine measurements were assessed by intracellular cytokine staining (ICS) with monoclonal antibodies specific for IFNγ (PerCp-Cy5.5, Biolegend) and TNFα (APC, Biolegend) and detected by FACS Canto II. In order to evaluate the activity of cytotoxic NK cells , CD107a degranulation marker was employed after PMA and ionomycin stimuli. Following the stimulus, the NK cells were incubated for 4 h with the antibody specific for CD107a (PE-Cy7 -BD) and the Golgi inhibitor, Brefeldin A (10 µg/ml). The results were displayed as the difference between cytokine- and CD107a-positive NK cells with or without stimulus. In order to study the adhesion ability of infiltrating NK cells toward K562 target cells, K562 cells were marked with a green fluorescent probe (CFSE, carboxyfluoroscein succinimidyl ester) and NK cells with antibodies specific for CD3 (APC-Cy7) and CD56 (APC-R700). To block CFSE staining, two volumes of cold fetal bovine serum were added, followed by washing thrice with Hanks’ balanced salt solution. The NK cells have been joined to target cells in a ratio of 1:5, respectively, and then they have been mixed by gentle vortexing and centrifuged for 3 min at 4°C (at 300 rpm). The cells were incubated at different timepoints (0, 15, and 30 min) at 37°C, fixed with 1 ml of Fixation Permeabilization Concentrate and Diluent (eBioscience), and then measured on FACS Canto II (BD). The NK cells which adhered to the K562 cells were analyzed, and the results were shown as mean fluorescence intensity related to the NK lymphocytes gated on target cells.

### Functional Restoration Assays

IFN-γ and TNF-α release from NK cells was assessed after IL-12 and IL-18 (5 ng/ml) overnight (O/N) stimuli in the presence or absence of specific p38 inhibitors. After 1 h of the stimulus, 10 µg/ml of BFA was supplemented for a further 3 h of culture. Anti-CD3 PE (BD), anti-CD56 PECF594 (BD), and anti-CD16 FITC (BD) were employed to stain the NK cells before fixing them with the medium A reagent and permeabilization with medium B reagent (Nordic Mubio) according to the manufacturer’s instructions. Cytokine production measurements were assessed by ICS after staining with monoclonal antibodies for IFNγ (PerCp-Cy5.5, Biolegend) and TNFα (APC, Biolegend) and subsequently run on FACS cytometry. The cytotoxic activity of NK cells was monitored through CD107a degranulation staining. Specifically, IL-12 and IL-18 were employed to stimulate the inhibitor-treated NK cells overnight (as described above). The cells together with target cells (K562) were then incubated with BFA and CD107 antibody (PECy7, BD) for the last 4 h.

The results are displayed as a ratio (fold change) of cytokine-positive NK cells detectable in inhibitor-treated * vs *. untreated cells. As control, the percentage of dead cells was evaluated in each inhibitor-treated sample by FACS in order to assess the cell toxicity.

### Metabolic Restoration Assays

Mitochondrial membrane potential (JC-1), glucose uptake, and autophagy potential by NK cells from LINK and TINK samples were evaluated, as described above, after O/N IL-12 + IL-18 (5 ng/ml) stimulation with or without the specific p38 inhibitors.

Data were presented as the ratio between the cells in the fluorescent channel FL1high and FL2low (JC-1 staining) and MFI (2-NBDG or Cyto-ID staining) detected in inhibitor-treated * vs *. untreated cells (fold change). As control, the percentage of dead cells was evaluated in each inhibitor-treated sample by FACS in order to assess cell toxicity.

The compounds tested were the SB203580 p38 inhibitor, used at 0.05–1 μM (Sellekchem), and doramapimod (BIRB-796), used at a concentration of 0.05–1 μM (Selleckem).

All assays were assessed on 8-color flow cytometer (FACS Canto II, BD), and the results were analyzed with FACS Diva and Flowjo software (BD). The frequency of CD56^DIM^ and CD56^BRIGHT^ NK cells was determined on the CD3-negative CD56-positive cells by evaluating the fluorescence intensity level of CD56 marker, and each parameter expression (carried out as percentage and MFI) was measured on total CD56 positive, CD56^DIM^, or CD56^BRIGHT^ NK cells .

### Statistical Analysis

Statistics was assessed with GraphPad Prism v.7 software. Analyses of variance (*F* test) and of normality (Kolmogorov–Smirnov test) were initially evaluated. Subsequently, Mann–Whitney *U*-test, paired *t*-test, Wilcoxon matched-pairs test, and Pearson’s correlation were used. Moreover, inhibitory drug experiments and fold change upon inhibitory treatments were statistically calculated by Wilcoxon signed rank tests (as compared to a theoretical median of 1).

All statistics were two-tailed, and significance was observed as *p* <0.05.

## Results

### Gene Expression Profiling of Tumor-Infiltrating NK Cells

**Figure 1:**
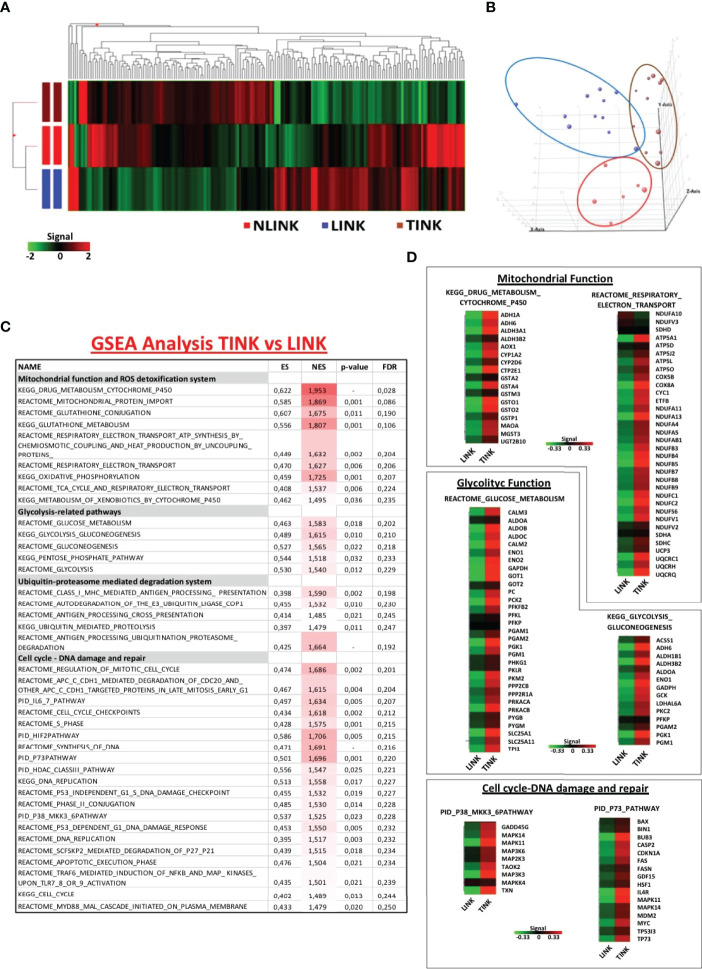
Gene expression pattern of infiltrating NK cells. **(A)** Hierarchical clustering representation of the 181 genes identified as differentially expressed in tumor-infiltrating NK cells (TINK, *n *= 11), surrounding liver-infiltrating NK cells (LINK, *n *= 11), and normal liver-infiltrating NK cells (NLINK, *n *= 7) by ANOVA with Benjamini–Hochberg correction (*p *≤ 0.05). Data were median-normalized before clustering; upregulated and downregulated genes are shown in red and green, respectively. **(B)** Principal component analysis of ANOVA-filtered data. **(C)** Enriched gene sets in TINK and LINK identified by Gene Set Enrichment Analysis (GSEA; MSigDB, C2 canonical pathways). NES, normalized enrichment score; FDR, false discovery rate. **(D)** Heat map of differentially expressed genes derived from GSEA in TINK and LINK related to mitochondrial and glycolytic function, cell cycle, and DNA damage/DNA repair. The upregulated genes are presented in red and the downregulated genes in green.

NK cells (CD3^-^, CD56^+^) were purified by flow cytometric cell sorting from tumor and liver tissue samples of patients with HCC arising in HCV infection (LINK and TINK, *n* = 11) and from liver control tissue samples (NLINK, * n * = 7). The analysis of variance on data of the detected probes (19,716 genes) identified 108 DEGs in NK cells from HCCs and controls. The hierarchical clustering of DEGs is depicted in **Figure 1A**. PCA showed a partial segregation among groups of patients and controls (**Figure 1B**).

We then focused on the comparison of NK cells infiltrating the HCC and the liver from the same patients and interrogated the Molecular Signatures database (http://www.broadinstitute.org/gsea). GSEA was conducted to understand the NK cell expression modifications in the tumor microenvironment. Gene sets related to mitochondrial functions and ROS detoxification system, glycolytic activity, ubiquitin-proteasome-mediated degradation system, and pathways related to cell cycle and DNA damage and repair (included the stress sensor p38-related pathway) appeared to be enriched and upregulated in TINK compared to LINK, as shown in **Figure 1C**.

Genes encoding the electron transport chain components and many subunits of complex I (NADH dehydrogenase), II (succinate dehydrogenase), III (cytochrome c reductase), IV (cytochrome c oxidase), and V (ATP synthase) were upregulated in HCC-infiltrating NK cells . Among the genes upregulated in tumor-infiltrating NK cells were genes related to glucose metabolism, particularly the aldehyde dehydrogenase family, aldolase complex, and phosphoglycerate kinase 1 (**Figure 1D**). Another group of transcripts upregulated in TINK encodes intracellular signaling and cell cycle control genes, such as MAPK14 and MAPK11 (subunits of p38 protein complex), TP53, p73, and p21 (**Figure 1D**).

Moreover, genes encoding the key components of the immune ubiquitin-proteasome pathway, including the 19S and 11S regulatory particles, the 20S proteolytic core, and the 26S proteasome subunits, resulted as upregulated in TINK. The detailed lists of genes may be found in **Supplementary Table S2**.

GSEA was also applied for the comparison between gene expression profiles of TINK and NLINK, leading to the identification of enriched upregulated genes related to the similar pathways described above in the previous comparison (**Supplementary Table S3**), confirming that tumor-infiltrating NK cells showed a misregulation in transcriptome profiles. The NK cells from tumor and liver tissue samples were analyzed by distinguishing the total NK cells from the CD56^DIM^ and CD56^BRIGHT^ subsets. A generalized upregulation thus appears to be the predominant feature of tumor-infiltrating NK cells .

### Impaired Mitochondrial and Glucose Metabolism of Tumor-Infiltrating NK Cells

Because of similar data reported for tumor microenvironment (32), we hypothesized that HCC-infiltrating NK cells are in a condition of nutrient deprivation, which is required to meet the increased energy and biosynthetic demands for NK cell expansion and anti-tumor functions (37, 38). To evaluate the possible intra-tumor dysregulation of NK cell metabolism, we first focused on mitochondrion and glucose metabolism.

**Figure 2:**
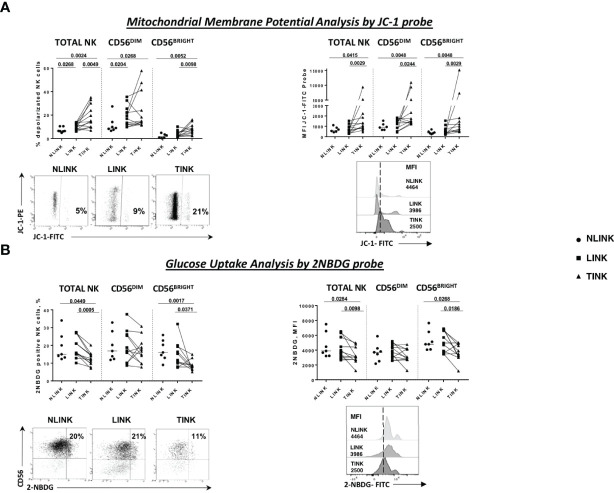
Metabolism assessment of infiltrating NK cells. **(A)** Mitochondrial membrane potential measured by the potentiometric probe JC-1. After anti-CD3 and anti-CD56 staining, JC-1 was added to NK cells from the three compartments: NLINK (*n *= 7), LINK (*n*= 12), and TINK (*n *= 12). Afterwards, the cells were stained with 7-AAD for viability and then analyzed on a flow cytometer. Depolarized NK cells (upper left panel) were quantified by the percentage of FL1high/FL2low cells (JC-1 staining) detected in the different samples. Median fluorescent intensity (MFI) of JC-1 in the FITC channel were analyzed in all study groups in the NK cells subsets (TOTAL, CD56^DIM^, and CD56^BRIGHT^). In the lower panels, representative examples of the two analyses. **(B)** Glucose uptake assay was performed on LINK (*n *= 12), NLINK (*n *= 7), and TINK (*n *= 12). The cells were stained with the glucose analog 2-NBDG. The frequency of 2-NBDG-positive NK cells was evaluated (upper left panel) as well as the MFI of the probe (upper right panel). Representative dot plots and histograms show the 2-NBDG uptake in NK cells. Statistical analysis was performed by Wilcoxon matched pairs test (LINK *vs*. TINK) and Mann Whitney test (NLINK *vs*. TINK and NLINK *vs*. LINK). Horizontal lines represent median values.

Mitochondrial membrane depolarization and glucose import were measured. As shown in **Figure 2A**, tumor-infiltrating NK cells displayed a defective mitochondrial functionality, indicated by increased depolarization of the mitochondria compared to the non-tumorous counterpart and the controls. On the left side is the frequency of JC-1-positive cells, while on the right side is the median fluorescence intensity, showing that tumor-infiltrating NK cells exhibited a higher content of depolarized mitochondria in all NK cell subsets (TOTAL, DIM, and BRIGHT).

Glucose uptake capacity, described by the 2-NBDG analysis, was reduced in TINK compared to NLINK and LINK cells, both in terms of the percentage of metabolically active NK cells and in terms of fluorescence intensity, confirming the existence of metabolic impairment at different levels (**Figure 2B**). CD56^BRIGHT^ TINK showed the worst performance in glucose uptake capacity.

### Stress Sensor p38 Kinase in Tumor-Infiltrating NK Cells

Then, we tried to clarify the mechanisms responsible for glycolytic impairment and to understand the upregulation of OXPHOS gene that was functionally ineffective. To this end, we performed a leading edge on GSEA data sets in order to extract a candidate list of regulatory genes. Notably, the majority of GSEA misregulated pathways highlighted an upregulation of MAPK11 and MAPK14 (α and β subunits of p38 complex).

**Figure 3:**
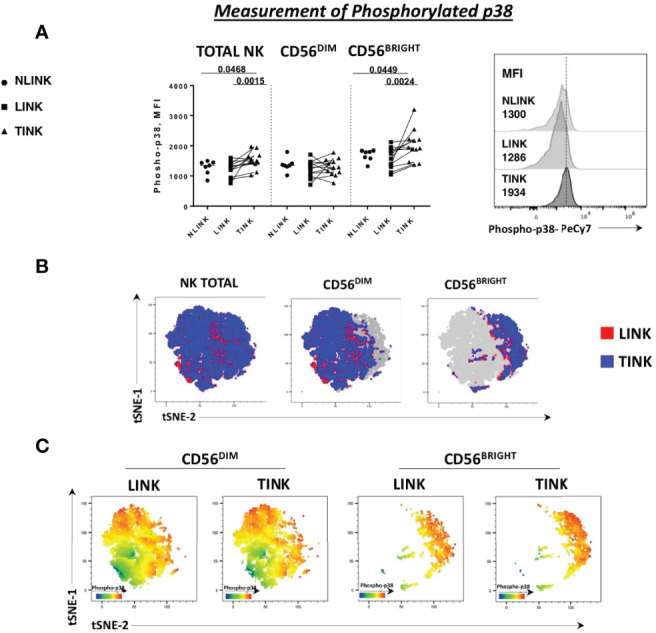
Phosphorylation status of p38 protein in NK cells infiltrating hepatocellular carcinoma (TINK), HCC-surrounding liver (LINK), and normal liver tissue (NLINK). **(A)** On the left, median fluorescence intensity (MFI) of phospho-p38 protein in total, CD56^DIM^, and CD56^BRIGHT^ NK-cell subpopulations from TINK (*n *= 12), LINK (*n *= 12), and NLINK (*n *= 7). Right panel: detail of phospho-p38 MFI values of total NK cells from different study groups. Statistical analysis was performed by Wilcoxon matched pairs test (LINK *vs*. TINK) and Mann-Whitney test (NLINK *vs*. TINK and NLINK *vs*. LINK). **(B)** To show the segregation of NK subsets, we generated a two-dimensional map of NK cells from paired TINK and LINK from all the experimental samples. tSNE was applied to flow cytometry data (single-cell expression values). **(C)** tSNE colored by the expression intensity of phospho-p38 protein in TINK and LINK samples. Red indicates upregulation, while blue indicates downregulation. Different color shades represent intermediate levels.

Indeed p38 protein is a stress sensor kinase from MAPK family that can be activated by nutrient deprivation in the microenvironment. Based on this observation, we analyzed the phosphorylation status of p38-MAPK protein in the three study groups. A higher level of phosphorylated p38, observed as median fluorescence intensity, was detected in total NK cells derived from tumor samples and was particularly enhanced in the CD56^BRIGHT^ subset (**Figure 3A**). This observation confirms the activation of this specific pathway as indicated by the transcriptomic analysis.

A dimension reduction by t-distributed stochastic neighbor embedding (tSNE) on flow cytometry data of phosphorylated p38 MAPK (pT180/pY182) from the LINK and TINK groups was performed to represent the relative intensity of NK-cell p38 expression. Despite the fact that the distribution of events was almost similar in the total, CD56^BRIGHT^, and CD56^DIM^ subsets in the two groups (**Figure 3B**), the color shades representing the protein level of p38 marker showed a higher expression in CD56^BRIGHT^ cells from the TINK samples (red indicating upregulation and blue indicating downregulation, **Figure 3C**).

### Autophagy Deregulation in Tumor-Infiltrating NK Cells

In order to better understand the metabolic assessment of HCC-infiltrating NK cells , the autophagy potential was analyzed.

The results of the transcriptomic analysis and validation suggested an engulfment of ROS clearance and accumulation of damaged mitochondria (**Figures 1C, D**, **2A**). In addition, p38 has been implicated in autophagy repression in different cell types, including T cells, during starvation conditions such as those occurring in the tumor microenvironment (39–41).

Similarly, we studied the lysosome degradation pathway by evaluating Cyto-ID cationic incorporation into autophagic vesicles (pre-autophagosomes, autophagosomes, and autophagolysosomes) with or without chloroquine treatment.

**Figure 4:**
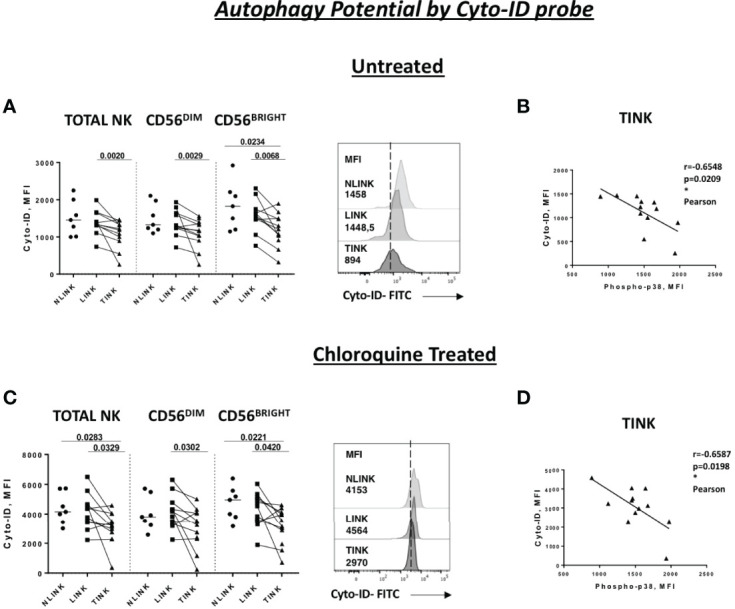
Autophagy capacity in tumor-infiltrating NK cells (TINK), non-tumorous liver-infiltrating NK cells (LINK), and normal liver-infiltrating NK cells (NLINK). Cyto-ID median fluorescence intensity (MFI) values in TINK (*n * = 12), LINK (* n * = 12), and NLINK (* n * = 7) from untreated **(A)** and chloroquine-treated **(C)** samples (total, CD56^DIM^, and CD56^BRIGHT^ NK cells). Examples are shown on the right of each panel. Statistical analysis was performed by Wilcoxon matched pairs test (LINK *vs * TINK) and Mann–Whitney test (NLINK *vs* TINK and NLINK *vs* LINK). Horizontal lines represent median values. Correlation between the Cyto-ID MFI values of untreated **(B)** and chloroquine-treated **(D)** and phospho-p38 protein in total NK cells from TINK samples. Statistics by Pearson correlation. *p < 0.05.

As presented in **Figure 4A**, TINK displayed a significantly lower basal Cyto-ID expression in all NK cell subsets compared to LINK and a limited one to CD56^BRIGHT^ compared to NLINK. After treatment with chloroquine, the reduced accumulation of autophagic vesicles was even more evident in HCC-infiltrating NK cells compared to NLINK and LINK (**Figure 4C**).

Cyto-ID expression was negatively correlated to p38 levels in TINK samples, suggesting a strong relationship between p38 and autophagy repression in TINK (**Figures 4B, D**).

### Functional Impairment of Infiltrating NK Cells

**Figure 5:**
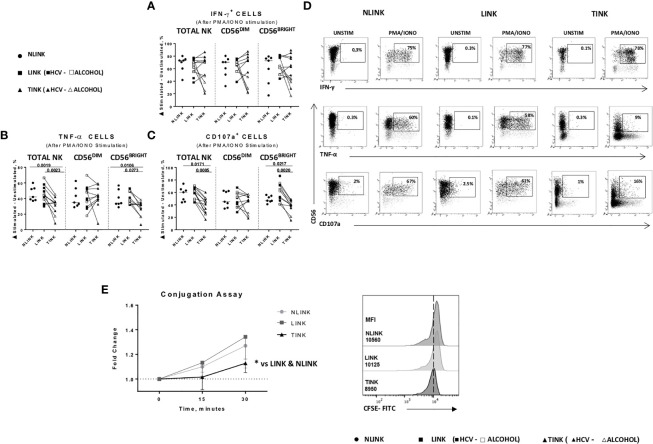
Functional analysis of NLINK, LINK, and TINK. **(A, B)** IFN-γ and TNF-α production in infiltrating NK cells was evaluated with or without phorbol 12-myristate 13-acetate and ionomycin incubation for 4(h) **(C)** The cytotoxic potential of NK cells was measured by CD107a degranulation assay. **(D)** Representative dot plots showing IFN-γ, TNF-α, and CD107a expression in tumor- and liver-infiltrating NK cells from NLINK, LINK, and TINK upon PMA/ionomycin stimulation. **(A**–**C)** Data are presented as the delta between the frequency of cytokine^+^ or CD107a^+^ NK cells in unstimulated and stimulated samples. Horizontal lines indicate median values. **(E)** NK cell adhesion to target cells (K562) was evaluated by staining NK cells and K562 with two distinct fluorescent dyes (anti-CD3 and anti-CD56 and 0.5 μM CFSE, respectively). The effector-to-target ratio was 5:1. Cells were incubated at 37°C for 0, 15, and 30 min, followed by fixation. Median fluorescence intensity (MFI) ratio was measured at different time points (left panel). Cell conjugation is presented as MFI values of CD56^+^ cells in CFSE-FITC target cells. Comparison of TINK, NLINK, and LINK at 30 min (right panel). **(A**–**E)** NLINK (*n* = 7), LINK (*n* = 12), and TINK (*n* = 12) *p < 0.05.

In order to define the functional capacity of tumor-infiltrating NK cells, cytokine production and degranulation (CD107a expression) activity were assessed. IFN-γ production was not significantly impaired in TINK compared to LINK and NLINK (**Figure 5A**).

Conversely, TNF-α (**Figure 5B**) and CD107a (**Figure 5C**) expression was significantly downregulated in TINK compared to liver-infiltrating NK cells (LINK and NLINK) (**Figure 5D**), particularly in the CD56^BRIGHT^ subset.

Dysfunctional NK adhesion to target K562 cells was detected in TINK, also suggesting an impairment in immunological synapse establishment by intratumor NK cells compared to NLINK and LINK (**Figure 5E**).

We then investigated the possible relationship between cytokine production/degranulation and metabolic functions (**Supplementary Figure S1**). IFN-y, TNF-α, and CD107a expression did not correlate to the frequency of NK cells with depolarized mitochondria and autophagy function (**Supplementary Figure S1**, plots on the left) in LINK, whereas glucose uptake turned out to be positively correlated with IFN-y production (**Supplementary Figure S1B**).

By contrast, the metabolic status of intratumor NK cells showed a strong association with cytokine production and degranulation capacity. In particular, the frequency of NK cells with depolarized mitochondria exhibited a negative correlation with respect to IFN-y, TNF-α, and CD107a expression (**Supplementary Figure S1D**) suggesting that the mitochondrial functionality in TINK impacts on cytokine production and cytotoxic function. Glucose consumption and autophagy potential were also strictly connected to the immune functions of HCC-infiltrating NK cells (**Supplementary Figures S1E, F**).

### Functional and Metabolic Restoration of Tumor-Infiltrating NK Cells by Targeting p38 Protein

**Figure 6:**
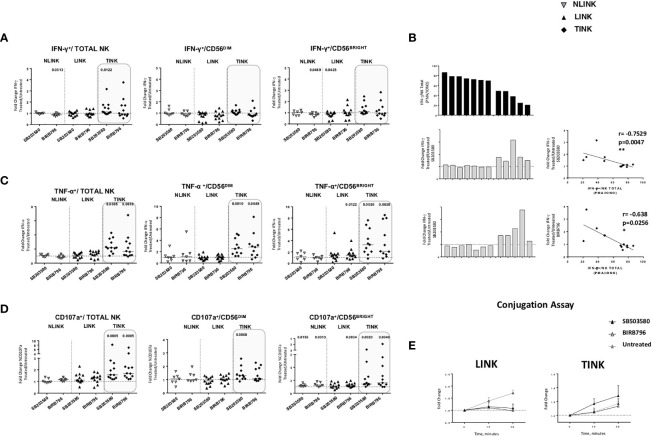
Restoration assays of hepatocellular carcinoma-infiltrating NK cells. IL-12 + IL-18 O/n stimulation of NK cell stimulation with or without specific p38 inhibitors was followed by flow cytometry determination of IFN-γ **(A)**, TNF-α **(B)** and CD107a **(C)** production in study groups and in all NK cell subsets (total, CD56^DIM^, and CD56^BRIGHT^). Data are presented as the ratio between the frequency of cytokine and CD107a-positive NK cells tested in the presence of inhibitor or untreated cultures (fold change). Horizontal lines represent median values. **(D)** Bar graph showing IFN-γ production upon PMA/ionomycin stimulation from single TINK samples (upper panel). On the middle and lower panels, corresponding fold change values in the two p38 inhibitor-treated TINK samples. Right panels: correlation between IFN-γ production and response to p38 blockade. **(E)** Conjugation assay performed on LINK and TINK samples with or without p38 inhibition. **(A–C)** Statistical analysis by Wilcoxon signed-rank tests.**(D)** Statistics by Pearson’s correlation. **(A–E)** LINK (*n*= 11) and TINK (*n*= 11). *p < 0.05, **p < 0.01.

To further analyze the relationship between p38 activation and TINK dysfunction, we assessed the effect on NK metabolic profile and anti-tumoral functions of two different chemical inhibitors targeting p38 (**Figures 6A–D**).

p38 blockade strongly increased the TNF-α (**Figure 6C**) and CD107a (**Figure 6D**) expression in TINK in all the NK subsets, whereas the effect on IFN-γ production was limited (**Figure 6A**). LINK and NLINK function did not benefit from p38 blockade, demonstrating the specificity of p38 inhibition in the tumor context. Representative dot plots showing CD107a, IFN-γ, and TNF-α expression in NK cells from NLINK, LINK, and TINK in untreated *vs*. p38 inhibitor-treated samples are shown in **Supplementary Figure S2**.

**Figure 7:**
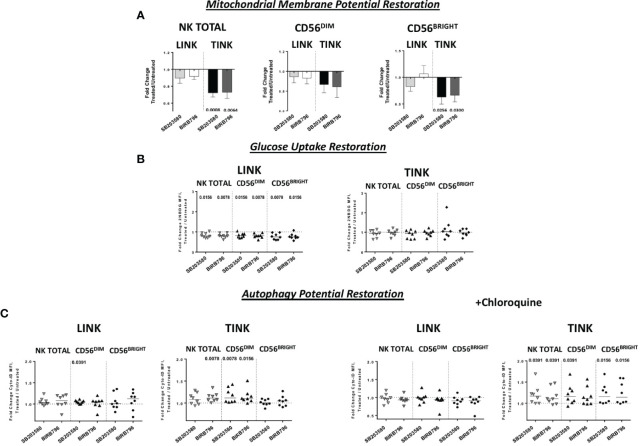
Metabolic restoration of HCC (*n* = 8) and non-tumorous liver-infiltrating NK cells (*n* = 8). IL-12 + IL-18 overnight stimulation with or without specific p38 inhibitors was followed by flow cytometry determination of JC-1 **(A)** 2-NBDG **(B)** and Cyto-ID (with and without chloroquine) **(C)**. Data are presented as the ratio between the metabolic values in inhibitor-treated *vs*. untreated NK cells from liver and tumor counterparts (fold change). Horizontal lines represent median values. Statistics by Wilcoxon signed-rank tests.

Interestingly, although IFN-y production was less affected than the other functions, TINK samples with impaired function were more affected by p38 inhibition (**Figure 6B**). Moreover, treatment with p38 inhibitors enhanced the adhesion to target cells by TINK compared to LINK, but the effect did not reach statistical significance (**Figure 6E**). Finally, p38 blockade strongly reduced the NK cells with depolarized mitochondria in the tumor, in particular in the CD56^BRIGHT^ subset, while p38 inhibition did not show any effect in modulating the mitochondrial membrane potential in the liver counterpart (**Figure 7A**). In addition, the NK autophagy potential was significantly enhanced by p38 inhibition, especially in the chloroquine-treated tumor-infiltrating NK cells (**Figure 7B**) in all NK cell subsets. Differently though, glucose import was not affected by treatment both in liver- and tumor-infiltrating NK cells (**Figure 7C**).

## Discussion

The understanding of the molecular and cellular characteristics of NK cell responses in patients with HCC has greatly improved in recent years (7, 11, 12). However, our knowledge of the mechanisms responsible for NK cell dysfunction in tumors and especially in HCC remains largely incomplete. Here we performed transcriptome, metabolic, and functional analyses targeting the identified dysregulated pathways of tumor-infiltrating NK cells . We found a predominantly upregulated gene expression profile in intra-tumoral NK cells compared to the liver counterpart. However, this extensive upregulation, involving mitochondrial components, glucose metabolism, and DNA damage stress responses, was not associated with an improved antitumor function but rather with a profound impairment at both functional and metabolic levels. This discrepant transcriptional and functional metabolic output was especially clear for OXPHOS impairment, with an altered mitochondrial membrane potential, and for glucose metabolism, associated with a decreased glucose uptake capacity. OXPHOS was upregulated at the transcriptional level but functionally depressed, and glucose use and glycolytic functions were similarly upregulated at the mRNA level but translated into an energetic repression in HCC-infiltrating NK cells .

A similar discrepancy between metabolic, functional, and gene expressions that could reflect a failed compensatory attempt has been described in exhausted CD8+ T cells (42). In particular, defective OXPHOS and glycolytic activity have recently been described in several different models (33, 43), including early-exhausted CD8+ T cells reported in HCV infection (44).

Even if we do not have clear direct evidence, we can imagine that, in tumors as well as in chronic infections, NK cells may present an exhausted condition similar to exhausted T cells, with poor effector characteristics (45), and a progressive loss of function correlating to disease progression.

Even if NK cell metabolism studies are in an early phase, T and NK cells have common metabolic pathways for several functions from activation to regulatory function (46).

Indeed both cell types become metabolically quiescent in mature status, using mainly fatty acid β-oxidation and oxidative phosphorylation when a change in energetic needs is required (46). Although metabolic needs may be different upon activation by activating ligands, a common feature seems to be the use of glucose for enhanced glycolysis and OXPHOS. This metabolic shift is dictated by an increase of energy demands within a short period of time for ATP and protein synthesis in order to accomplish their cytotoxic functions (46).

Our GSEA results support the mechanism showing an upregulation of ROS detoxification system-related pathways. In line with this view, our data show an increase of depolarized mitochondria in HCC-infiltrating NK cells and a decrease in potential glucose utilization, especially in the CD56^BRIGHT^ subpopulation, translated into an inefficient cytotoxic activity, as supported by the downregulation of TNF-α and CD107a expressions and by a poor ability to establish an immunological synapse with target cells (44).

While the production of inhibitory cytokines in the tumor microenvironment induces a profound immune impairment, immune cells react by activating the stress sensor-related pathways. Specifically, cellular stress induced by starvation leads to MAPK/p38 cascade activation. p38 subunits (such as p38α and MAPK14), after activation and phosphorylation, control a plethora of downstream functions. In particular, MAPK14 is responsible of the quick change from glycolysis to the pentose phosphate pathway (PPP) through modulation of the proteasome degradation of 6-phosphofructo-2-kinase/fructose 2,6-bisphosphatase 3. This leads to reduced autophagy and an increase in resistance to nutrient starvation (47). The results of NK-cell expression profiling support this view, suggesting the activation of the PPP pathway with an upregulation of PFK genes.

Under nutrient stress, there is activation of the catabolic process of autophagy with turnover of macromolecules and organelles (48). Since autophagy is considered a mechanism of cell survival, its deregulation does not lead to apoptotic cell death. It is known that a robust autophagic capacity appears in immature NK cells in order to lead to NK cell development and education (33, 49). During development, autophagy protects NK cells by removing damaged mitochondria that reduce the ROS levels (33).

It has already been described for CD8+ T cells that, under low nutrient conditions and high proliferative rate, p38 is phosphorylated *via* the AMP-activated protein kinase (AMPK) and the scaffold protein β-activated TAB1 (50). This leads to a metabolic reprogramming with glycolysis and autophagy reduction and with consequent blocking of mitophagy and accumulation of damaged and dysfunctional mitochondria. The final effect of p38 activity augmentation is metabolic engulfment and proliferation arrest (40, 51), which are associated to a senescent-like behavior with a negative effect on telomerase activity (40, 50). Similarly, in a previous study, we found that intra-tumoral NK cells show a profound reduction of intracellular transducers, including the proximal CD3 zeta chain (34, 52), and our current analysis also confirms a deregulation in the autophagy process related to p38 activation in tumor-infiltrating NK cells.

However, long-lasting activation of the p38α pathway can lead to impaired cell proliferation and cell death (53), which might explain the lower level of mature and cytotoxic intratumor NK cells (52). These profound metabolic derangements impair the capacity of NK cells to exert efficient anti-tumor functions (54) and to generate immunological memory, together with a lower basal level of autophagy, which are essential in NK cell development (33, 49). Targeting p38 may open to new strategies of immunotherapeutic intervention.

Interestingly, the CD56^BRIGHT^ subset showed a higher level of p38 phosphorylation and functional impairment. This subset expresses liver residency markers such as CXCR6 and CD49a (52) at a higher degree, and it is easy to speculate that the NK cells in HCC have been recruited from the liver or from the periphery, and in a hypoxic tumor environment, they upregulate CD56 (55). While the entire population of TINK is in a stress condition due to hypoxia and starvation, the CD56^BRIGHT^ subset has been more profoundly impaired.

Despite the lack of direct supporting evidence in NK cells , a direct relationship between p38 upregulation and metabolic and functional impairment in HCC-infiltrating NK cells is likely, as already described for CD8+ T cells (40, 50).

For this reason, the pharmacological inhibition of p38 protein subunits could be a therapeutic option in patients with early HCC. Our data indicate that p38 modulation is highly specific for tumor-infiltrating NK cells, thus not affecting the functions of NK cells infiltrating liver tissue. In tumor NK cells, we observed that p38 blockade induced a strong restoration of TNF-α and CD107a production accompanied by increased IFN-γ expression in samples with a reduced production of this cytokine. These results suggest that NK cells might be exhaustion-oriented at different levels and that selective targeting of p38 might allow specific functional recovery. In addition, metabolic functions such as OXPHOS and autophagy were significantly improved by p38 inhibition, specifically in HCC-infiltrating NK cells, further indicating a positive impact of p38 blockade over NK cell fitness.

The results of this study are not sufficient to indicate a specific biochemical metabolic cascade of dysregulation of HCC-infiltrating NK cells and to define how OXPHOS derangement and impaired autophagy are linked to mitochondrial dysfunction and p38 activation. Given the functional recovery obtained with selected specific p38 inhibitors, the knowledge generated in this study altogether has relevant potential for novel and more effective strategies of NK cell reconstitution, thus able to improve immunotherapy for HCC patients.

## Data Availability Statement

The original contributions presented in the study are publicly available. This data can be found here: https://www.ncbi.nlm.nih.gov/genbank/, GSE183349.

## Ethics Statement

The studies involving human participants were reviewed and approved by Comitato Etico Indipendente Area Vasta Emilia Nord, Italy. The patients/participants provided their written informed consent to participate in this study.

## Author Contributions

AZ: study concept and design, analysis, and interpretation of data, drafting of the manuscript, critical revision of the manuscript for important intellectual content, and statistical analysis. VB: acquisition of data, analysis and interpretation of data, and statistical analysis. AO: acquisition of data and technical or material support. EB: acquisition of data and technical or material support. CB: acquisition of data and critical revision of the manuscript for important intellectual content. PF: acquisition of data and critical revision of the manuscript for important intellectual content. IM: acquisition of data and technical or material support. CT: acquisition of data and technical or material support. RDV: acquisition of data, technical or material support, and critical revision of the manuscript for important intellectual content. CF: drafting of the manuscript and critical revision of the manuscript for important intellectual content. EC: study concept and design, drafting of the manuscript, and critical revision of the manuscript for important intellectual content. GM: study concept and design, drafting of the manuscript, critical revision of the manuscript for important intellectual content, obtained funding, and study supervision. All authors contributed to the article and approved the submitted version.

## Funding

This work was supported by a grant from Italian Association for Cancer Research (AIRC) IG 15485 and HUNTER-Accelerator Award 22794 (AIRC, CRUK, AECC).

## Conflict of Interest

AZ, VB and GM are inventors on patent filed, owned and managed by University of Parma on technology related to the work presented in this manuscript (IT patent application #IT102022000000314). CF received a grant from Gilead and Abbvie as well as serves as a consultant for Gilead, Abbvie, Vir Biotechnology Inc., Arrowhead, Transgene, and BMS.

The remaining authors declare that the research was conducted in the absence of any commercial or financial relationships that could be construed as a potential conflict of interest.

## Publisher’s Note

All claims expressed in this article are solely those of the authors and do not necessarily represent those of their affiliated organizations, or those of the publisher, the editors and the reviewers. Any product that may be evaluated in this article, or claim that may be made by its manufacturer, is not guaranteed or endorsed by the publisher.

## Supplementary Material

Supplementary Table 1 Demographic and clinical characteristics of study population.

Supplementary Table 2 Detailed lists of misregulated genes derived from Gene Set Enrichment Analysis.

Supplementary Table 3 Gene Set Enrichment analysis (GSEA). GSEA identified the list of enriched gene sets in TINK and LINK by MSigDB and C2 canonical pathways set. NES, normalized enrichment score; FDR, false discovery rate.

Supplementary Figure 1 Correlation between TINK and LINK metabolism and functions. **(A)** Correlation between the frequency of depolarized mitochondria in LINK (*n* = 12) total NK cells and corresponding IFN-γ, TNF-α, and CD107a production.**(B)** Correlation between glucose uptake, described by 2-NBDG median fluorescence intensity, in LINK and corresponding IFN-γ, TNF-α, and CD107a production. **(C)** Correlation between the frequency of CD107a+, IFN-γ, and TNF-α and the Cyto-ID MFI values in LINK samples. **(D–F)**. Correlation between the percentage of CD107a+, IFN-γ, and TNF-α with mitochondrial function **(D)** glucose uptake **(E)** and autophagy potential in TINK (*n*= 12) **(F)**. Statistics by Pearson’s rank-order correlation.

Supplementary Figure 2 Flow cytometry analysis of NK cell functional and metabolic restoration. **(A)** Representative dot plots showing IFN-γ, TNF-α, and CD107a expression in NK cells from NLINK, LINK, and TINK in untreated * vs *. p38 inhibitor-treated samples. **(B)** Representative dot plots showing the percentage of depolarized mitochondria in tumor- and liver-infiltrating NK cells in untreated *vs*. p38 inhibitor-treated samples. **(C)** Histograms representing glucose uptake modulation in treated *vs*. untreated samples in LINK and TINK samples. **(D)** Representative histograms showing Cyto-ID staining in NK cells from LINK and TINK in untreated *vs*. p38 inhibitor-treated samples.
