# Supplementary figures and images for: Targeting Stress Sensor Kinases in Hepatocellular Carcinoma-Infiltrating Human NK Cells as a Novel Immunotherapeutic Strategy for Liver Cancer
Source: Front Immunol. 2022 May 23;13:875072. doi: 10.3389/fimmu.2022.875072 (PMC9168800; doi:10.3389/fimmu.2022.875072)

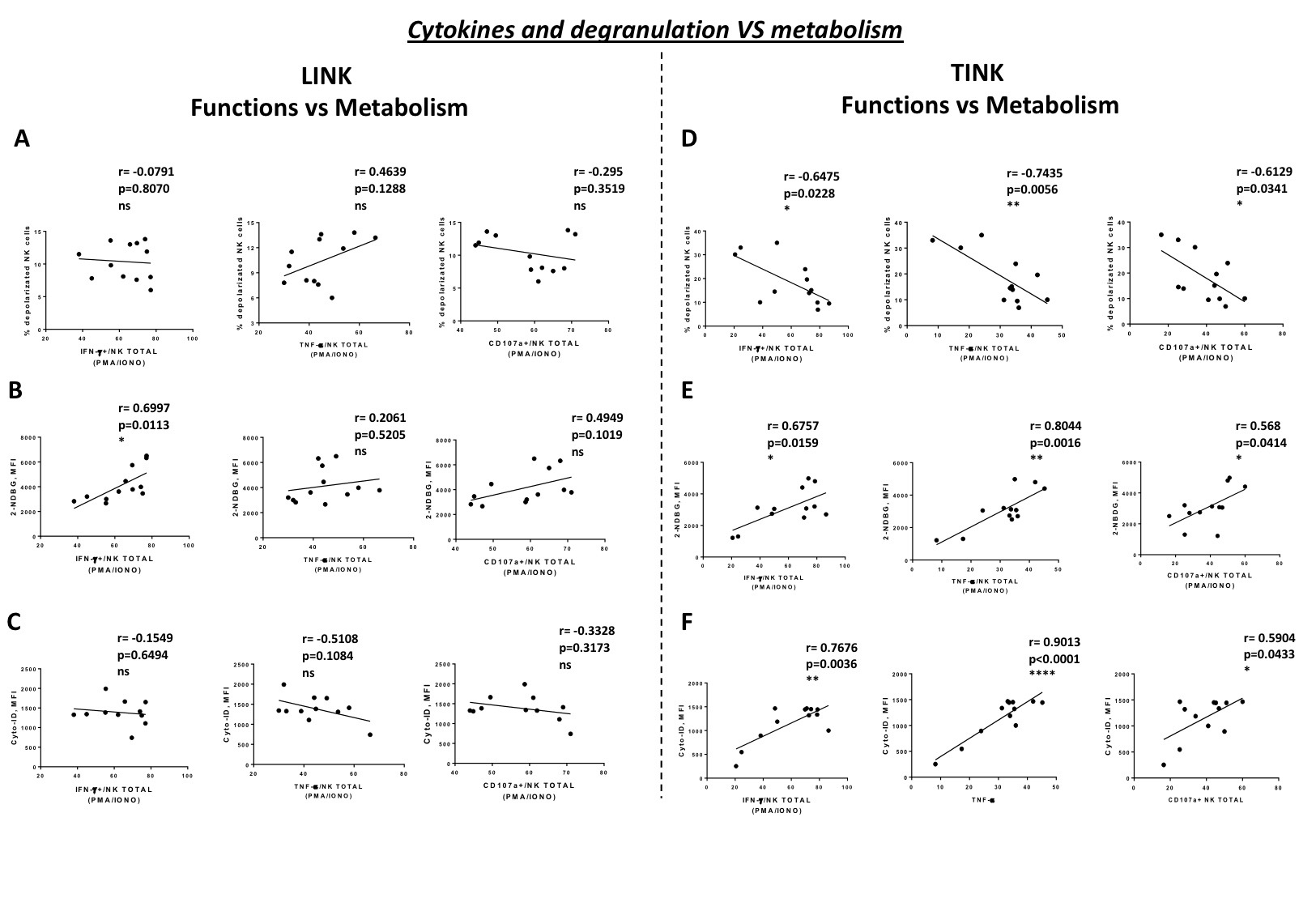

Supplement: Supplementary Figure 1 — Correlation between TINK and LINK metabolism and functions. (A) Correlation between the frequency of depolarized mitochondria in LINK (n = 12) total NK cells and corresponding IFN-γ, TNF-α, and CD107a production.(B) Correlation between glucose uptake, described by 2-NBDG median fluorescence intensity, in LINK and corresponding IFN-γ, TNF-α, and CD107a production. (C) Correlation between the frequency of CD107a+, IFN-γ, and TNF-α and the Cyto-ID MFI values in LINK samples. (D–F). Correlation between the percentage of CD107a+, IFN-γ, and TNF-α with mitochondrial function (D) glucose uptake (E) and autophagy potential in TINK (n= 12) (F). Statistics by Pearson’s rank-order correlation. [file Image_1.jpeg]

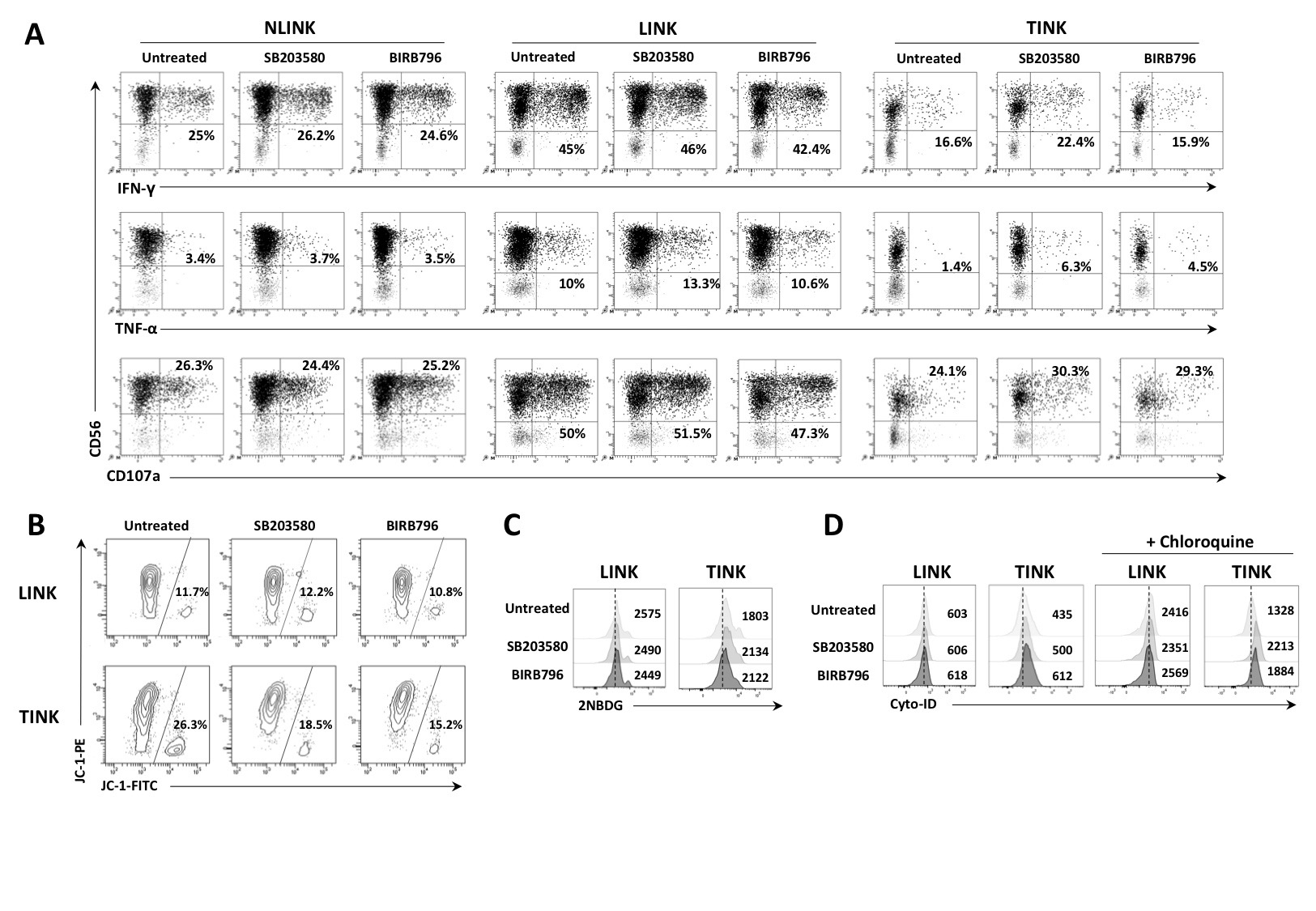

Supplement: Supplementary Figure 2 — Flow cytometry analysis of NK cell functional and metabolic restoration. (A) Representative dot plots showing IFN-γ, TNF-α, and CD107a expression in NK cells from NLINK, LINK, and TINK in untreated vs . p38 inhibitor-treated samples. (B) Representative dot plots showing the percentage of depolarized mitochondria in tumor- and liver-infiltrating NK cells in untreated vs. p38 inhibitor-treated samples. (C) Histograms representing glucose uptake modulation in treated vs. untreated samples in LINK and TINK samples. (D) Representative histograms showing Cyto-ID staining in NK cells from LINK and TINK in untreated vs. p38 inhibitor-treated samples. [file Image_2.jpeg]
